# Manifold role of ubiquitin in *Helicobacter pylori* infection and gastric cancer

**DOI:** 10.1007/s00018-021-03816-8

**Published:** 2021-04-07

**Authors:** Olga Sokolova, Michael Naumann

**Affiliations:** Medical Faculty, Otto Von Guericke University, Institute of Experimental Internal Medicine, 39120 Magdeburg, Germany

**Keywords:** E3 ubiquitin ligases, Bacteria, Inflammation, NF-κB, MDM2, p53, β-catenin, T4SS, Cancer

## Abstract

Infection with *H. pylori* induces a strong host cellular response represented by induction of a set of molecular signaling pathways, expression of proinflammatory cytokines and changes in proliferation. Chronic infection and inflammation accompanied by secretory dysfunction can result in the development of gastric metaplasia and gastric cancer. Currently, it has been determined that the regulation of many cellular processes involves ubiquitinylation of molecular effectors. The binding of ubiquitin allows the substrate to undergo a change in function, to interact within multimolecular signaling complexes and/or to be degraded. Dysregulation of the ubiquitinylation machinery contributes to several pathologies, including cancer. It is not understood in detail how *H. pylori* impacts the ubiquitinylation of host substrate proteins. The aim of this review is to summarize the existing literature in this field, with an emphasis on the role of E3 ubiquitin ligases in host cell homeodynamics, gastric pathophysiology and gastric cancer.

## Ubiquitin code

**Fig. 1:**
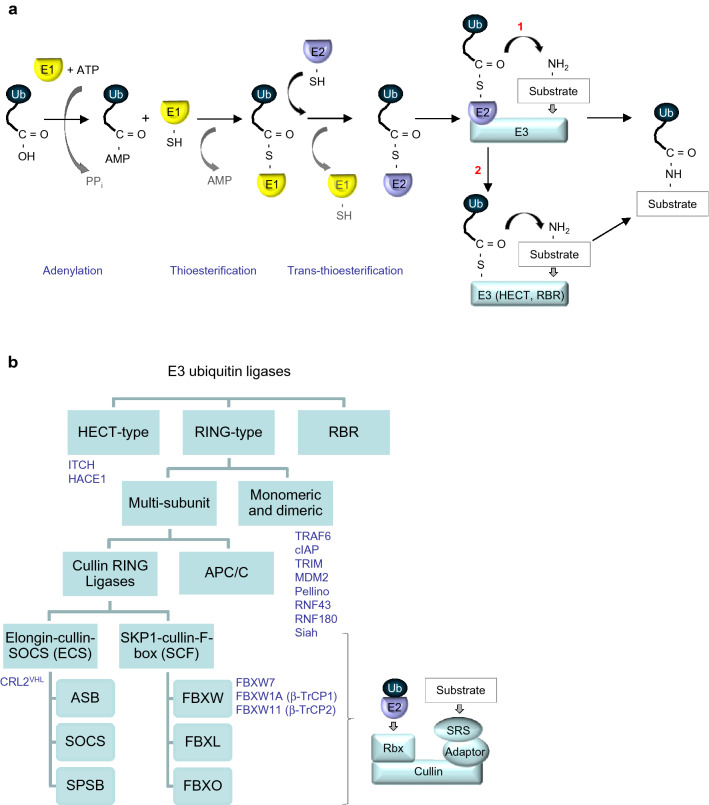
**a** Multistep process of ubiquitinylation. The glycine residue at the C-terminus of ubiquitin reacts with an internal cysteine residue of an ubiquitin-activating enzyme (E1) and, in follow, with a cysteine of an ubiquitin-conjugating enzyme (E2). The E2 interacts with an E3 ubiquitin ligase, which facilitates formation of a stable isopeptide bond between the glycine residue at the C-terminus of ubiquitin and a specific internal lysine residue of a substrate protein (1). HECT-type E3 ligases and RING-between-RING (RBR) E3 ubiquitin ligases interact with the E2 enzyme and form a thioester intermediate with ubiquitin before transfer it to a substrate (2). **b** Types of E3 ubiquitin ligases and composition of a multisubunit cullin-RING ligase. E3 ubiquitin ligases are represented by 3 main groups according to their structure and ability to react with ubiquitin. In the multisubunit RING-type E3s, adaptor proteins can be represented by Skp1 or elongin molecules. *SRS* substrate recognition subunit, *APC/C* the anaphase promoting complex/cyclosome, *Rbx* RING-box protein. The E3 ubiquitin ligases mentioned in the review are depicted

Much progress has been achieved in recent years in understanding the role of ubiquitinylation, which influences protein stability and function and thereby regulates a broad range of cellular processes. Ubiquitin, a 76-amino acid protein, can be attached via its glycine 76 residue to the ɛ-amino group of an internal lysine residue in a target protein via three steps: first, it forms a high-energy thioester linkage with a cysteine in the active site of an ubiquitin-activating enzyme (E1) in an ATP-dependent manner; second, it is further transferred to a cysteine in the active site of an ubiquitin-conjugating enzyme (E2); third, the ubiquitin-E2 complex interacts with an E3 ubiquitin ligase, which recruits a target protein via specific protein–protein interaction domains [1] (Fig. 1a).

Interestingly, the conjugation of other ubiquitin-like molecules, e.g., small ubiquitin-like modifier 1 (SUMO1), FAT10, ISG15, and NEDD8, to a protein substrate involves an analogous stepwise mechanism [2].

In humans, more than 600 E3 ubiquitin ligases have been described, and their diverse structures determine their selectivity towards ubiquitin acceptors. To date, E3 enzymes are classified into three main groups: homologous to the E6-associated protein carboxyl terminus (HECT) ligases (28 members), the really interesting new gene (RING) family (600 members) and RING-between-RING (RBR) family (14 members) [3] (Fig. 1b). HECT-type E3 ligases directly bind ubiquitin via their active site cysteine before they transfer it to the protein substrate. RING ligases lack a catalytic site, do not form thioester intermediates with ubiquitin and typically function as scaffolds and allosteric regulators. Features of the first two families are combined in the RBR family members for their sophisticated regulation: the RING1 domain of the RBR ligase recruits the E2-ubiquitin conjugate, and the catalytic cysteine in the RING2 domain binds a glycine in activated ubiquitin [4].

On a protein substrate, ubiquitin can form polypeptide chains where the C-terminus of one molecule binds to the methionine 1 (Met1) residue or one of seven internal lysines (6, 11, 27, 29, 33, 48 or 63) of a previously attached ubiquitin molecule. Such polyubiquitin chains can be assembled through homogeneous or heterogeneous linkage types; they differ in topology and length, and they determine the fate of the protein substrate. It has been proposed that monoubiquitinylation can alter protein localization, Lys48-linked ubiquitinylation targets protein substrates for proteasomal degradation, and Met1-linked (linear) or Lys63-linked polyubiquitinylation leads to interaction of the protein substrate with ubiquitin-binding domains (UBDs) of effector proteins and thereby triggers the formation of multimolecular protein nodes essential for cellular signal transmission [5]. The abundance of diverse ubiquitin chains, their distribution, and their specificity towards particular substrates and cellular processes remain poorly understood. It is also not clear whether the amino acid sequence surrounding the lysine residue in the protein substrate can determine ubiquitin chain binding and structure [6].

Ubiquitin adducts can be cleaved by deubiquitinylases (DUBs). In the human genome, there are approximately 100 DUBs, which have redundant activity, to some extent, towards different substrates; the target specificity of these DUBs is under intensive investigation. Generally, the high number of E3 ligases and DUBs, as well as the diversity of polyubiquitin chains make the ubiquitin signaling machinery very complex. However, mutations in particular DUBs and E3 ligases cause severe disorders. For example, mutations in CYLD, USP8, A20 and the von Hippel-Lindau tumor suppressor (VHL; elongin-cullin-2-VHL (CRL2^VHL^) E3 ubiquitin ligase) are related to cylindromatosis, Cushing disease, autoinflammatory syndrome and heritable cancer syndrome, respectively. These observations generate motivation to further investigate the ubiquitinylation process and to develop pharmacological tools for its therapeutic targeting [7].

## Bacteria and ubiquitinylation

In bacteria, two types of polypeptide modifiers interacting with a substrate lysine have been described: prokaryotic ubiquitin-like protein (Pup) in Actinobacteria (*Mycobacterium tuberculosis*, for example), which targets bacterial proteins for degradation, and bacterial ubiquitin-fold polypeptides small archeal modifier proteins (SAMPs) and *Thermus* tRNA-two-thiouridine B (TtuB), that differ from ubiquitin in sequence but share a common structural β-grasp fold [8]. There are no conventional E2 or E3 enzymes which would mediate ubiquitin-like post-translational modifications (PTMs) in bacteria, however, some prokaryotic enzymes are able to manipulate the eukaryotic ubiquitin system. Being secreted or injected into the host cell via their multiprotein secretion systems, the bacterial effectors can mimic the function of host E1, E2 or E3 enzymes, use an alternative ATP-independent mechanism or exhibit isopeptidase activity. Such enzymes were classified in several groups, including novel E3 ligases (NELs) (*Salmonella*’s SspH and *Shigella*’s IpaH proteins) and homologs to the eukaryotic HECT-type and RING-type E3 ligases (*Salmonella*’s SopA, NleL and NleG from pathogenic *Escherichia coli*) [8]. They can compete with the host E3 ligases for E2 binding and induce Lys48-linked ubiquitinylation and, thereby, cause the mislocalization and proteolysis of host proteins. For example, IpaH9.8 promotes ABIN-1-dependent ubiquitinylation of NF-κB essential modulator (NEMO) and its proteasome-dependent degradation [9]. Bacterial effectors can affect the host ubiquitinylation machinery by inducing inactivation of E1 and E2. For example, enteropathogenic *Escherichia coli* (EPEC) induce aspartyl protease-dependent degradation of E1 Ubc1 and UBA6, the only two E1 enzymes in mammalian cells, and cause a profound decrease in overall levels of ubiquitinylated proteins in HeLa cells [10]. *Shigella flexneri* protein OspI can deactivate E2 Ubc13 (UBE2N) through deamidation of its glutamine 100 and thus inhibit TNF Receptor-Associated Factor (TRAF) 6-NF-κB axis [11].

By influencing ubiquitinylation directly or indirectly, the bacterial effectors interfere with the key signaling pathways involved in the regulation of the cell cycle or innate immunity. Such strategy allows bacteria to mitigate inflammatory responses and to survive using the host cells as nutrients source.

For *Helicobacter pylori*, no bacterial effector has been described yet which would directly interfere with ubiquitin transferase reactions in host cells. However, the host cells infected with *H. pylori* demonstrate strong activation of ubiquitin-dependent signal transmission.

## Host cell protein ubiquitinylation in *H. pylori* infection and gastric cancer

*H. pylori* is a microaerophilic, gram-negative bacterium, which infects gastric mucosae of about two-thirds of the world’s human population. *H. pylori* is a main cause of gastritis, sometimes asymptomatic (www.CDC.gov). Prolonged infection with *H. pylori* is a risk factor for peptic ulcer disease and gastric carcinoma development, especially when combined with such factors as a salty diet, smoking, other chronic infections or host genetic predisposition (e.g., polymorphism in some genes). Despite intensive investigations in the field, gastric cancer remains one of the leading causes of cancer death worldwide, following lung cancer [12]. This reflects low-efficiencies of preventive diagnostics and therapy strategies, and further motivates for searching of new diagnostic markers and drug targets.

**Fig. 2:**
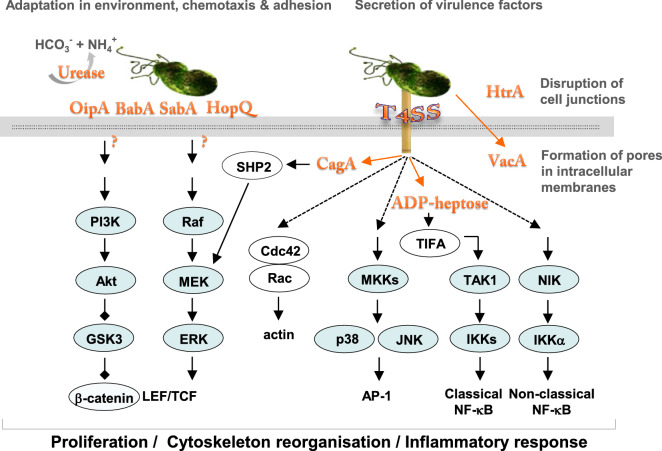
*H. pylori* virulence factors have impact on host cell signaling pathways. *H. pylori* secrete urease, which increases pH in the microenvironment and supports bacterial survival. A set of adhesins allow the bacteria to attach to the host cell surface and to build a T4SS. CagA and, putatively, ADP-heptose are translocated through the T4SS to the cytoplasm and activate SHP2 or ALPK1/TIFA, respectively. ADP-heptose triggers activation of the classical NF-κB pathway. MAP kinases JNK and p38 and the non-classical NF-κB signaling pathway are regulated in a T4SS-dependent but CagA-independent manner (dashed arrows). Bacterial factors responsible for activation of the Akt and MEK/ERK signaling pathways are not known, although an involvement of some adhesins has been discussed. The T4SS and CagA seem to be dispensable for these pathways. *H. pylori* secrets VacA, which internalizes into host cells, undergoes processing and assembles in stable hexamers that form an ion channel in intracellular membranes. It induces formation of giant vacuoles and, finally, cell death. A serine protease HtrA secreted by *H. pylori* cleaves E-cadherin and thereby is involved in disruption of adherens junctions and epithelial integrity during infection

*H. pylori* harbors a variety of virulence factors supporting adaptation of the bacteria to the environment and colonization of the human stomach. These include among others: urease, outer membrane proteins and adhesins, e.g., BabA, SabA, OipA, HopQ, vacuolating cytotoxin A (VacA), proteases, including HtrA, proinflammatory pathogen-associated molecular pattern (PAMP) molecules β-ADP heptose and the cytotoxin-associated gene pathogenicity island (cagPAI) gene products (Fig. 2). Most of the cagPAI-encoded proteins form a type 4 secretion system (T4SS), a pilus-like macromolecular transporter, which delivers cytotoxin-associated gene A (CagA), also a cagPAI gene product, into the cytoplasm of the host cell [13, 14]. CagA has been proposed to contribute to gastric carcinogenesis, but the mechanism is still under investigation. In the host cell, CagA interacts with a number of proteins via its intrinsically disordered C-terminal tail containing the Glu-Pro-Ile-Tyr-Ala (EPIYA) motifs and the CagA multimerization motif. It binds and deregulates Src homology 2 domain-containing protein tyrosine phosphatase 2 (SHP2) (Fig. 2) and polarity regulator partitioning-defective 1b (PAR1b) leading to changes in actin cytoskeleton dynamics [15]. *H. pylori* with functional CagPAI are strong inducers of the host kinases JNK1/2, p38, protein kinase C, Src and transcription factors AP-1 and nuclear factor κB (NF-κB) in gastric epithelium [16, 17]. The AP-1 and NF-κB, which drive the expression of proinflammatory molecules interleukin (IL)-1, IL-6, IL-8 and cyclooxygenase-2 (COX-2), represent a key defensive mechanism against infection agents. In addition to its role in innate immunity, NF-κB regulates expression of the pro-proliferative cyclin D1, p21, c-Myc, vascular endothelial growth factor (VEGF), epidermal growth factor receptor (EGFR), glucocorticoid receptor; anti-apoptotic Bcl-2, Bcl-X_L_, and cellular inhibitor of apoptosis (cIAP) proteins [18]. Long-lasting *H. pylori* infection together with host proinflammatory mediators, including TNF, IL-1, IL-6, and reactive oxygen species released by endothelial and immune cells, stimulate a net of signaling pathways and transcription factors, that finally accelerates a cell turnover in the targeted tissue. Together with a deficit in DNA repair, it promotes a carcinogenic transformation [19, 20].

It has been reported that an infection with *H. pylori* influences the host ubiquitin–proteasome system that is represented by the increase of ubiquitinylated proteins and reduction of DUB USP7 at the protein and transcript levels in the cell culture in a partially *cag*PAI- and CagA-dependent manner [21]. On the other hand, an increase of total ubiquitinylation (in AGS cells infected with *H. pylori* 26695) was not detected by Alvarez et al. [22]. Further, Necchi et al. found in gastric endoscopic biopsies from dyspeptic patients an accumulation of *H. pylori* virulence factors VacA, CagA, urease and outer membrane proteins together with E1, polyubiquitinylated proteins and proteasome components in particle-rich cytoplasmic structures (PaCS) [23]. Similar co-localization was found in infected human epithelial cell lines [23]. It has been suggested that the proteasome-enriched structures could modulate inflammatory and proliferative responses in the gastric epithelium. The proteasome activity has been found to support *H. pylori* adhesion to AGS cells. This mechanism involved a cullin-3 interactor (and perhaps a substrate adaptor) KCTD5, which was ubiquitinylated and further degraded in a CagA- and VacA-independent manner [22].

### E3 ubiquitin ligases TRAF6 and SCF^β−TrCP^ in NF-κB regulation

Major signaling pathways triggered by *H. pylori*, including the NF-κB, involve a set of E3 ubiquitin ligases and are strongly regulated by ubiquitinylation. The NF-κB signaling pathway participates in a broad spectrum of physiological and pathophysiological processes, such as cell proliferation, apoptosis, innate and adaptive immune responses and tumorigenesis [24]. In mammals, the NF-κB transcription factors family consists of five members known as RelA, RelB, c-Rel, p50/p105 (NF-κB1), and p52/p100 (NF-κB2). All the members contain an N-terminal domain of about 300 amino acids known as the Rel-homology domain (RHD), which mediates DNA binding and dimerization. The NF-κB subunits can form homo- or heterodimers in vivo. The Rel subfamily RelA, RelB and c-Rel are associated with one of the inhibitor of κB (IκB) proteins: IκBα, IκBβ or IκBε, and thereby retained in the cytoplasm in unstimulated cells [25].

p50 and p52 are translated as large precursor proteins p105 and p100, respectively. The C-terminal regions of p105 and p100 contain ankyrin repeats important for protein–protein interaction. The folding of the ankyrin domain masks the nuclear localisation sequence within N-terminal part of p100 and p105, making these proteins to be localized in the cytoplasm of unstimulated cells, where they, similar to IkBs, bind and sequester Rel proteins [26].

**Fig. 3:**
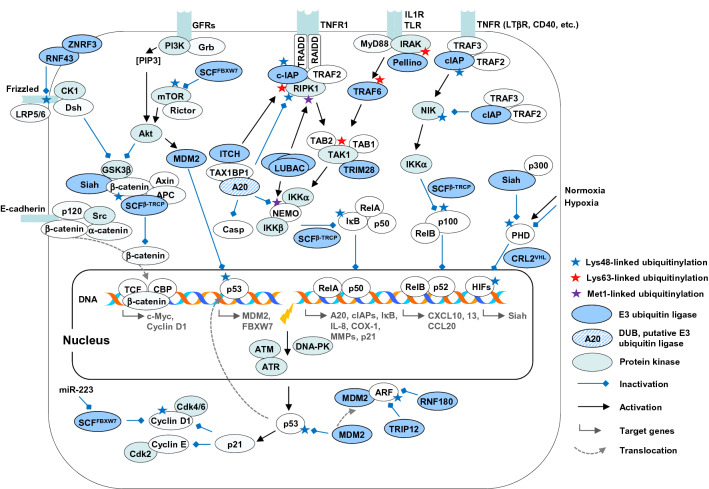
E3 ubiquitin ligases in signaling pathways related to *H. pylori* infection and gastric cancer (detailed description is in the main text). *GFRs* growth factor receptors, *TNFR* TNF receptor, *IL1R* IL-1β receptor, *TLR* Toll-like receptor

Although NF-κB subunits are ubiquitously expressed, they are regulated in a stimulus-specific and cell/tissue-specific manner. The classical and the non-canonical pathways trigger the activation of NF-κB transcriptional activity (Fig. 3). The classical pathway is induced in response to various inflammatory stimuli, such as cytokines TNF and IL-1β, the T-cell receptor (TCR) ligands or the PAMPs, such as bacterial lipopolysaccharide (LPS) and β-ADP heptose [27, 28]. Upon stimulation, diverse receptor-proximal molecular complexes are activated, including TNF receptor-associated protein with death domain (TRADD)/TRAF2/receptor-interacting protein kinase 1 (RIPK1) or MyD88/TIR Domain Containing Adaptor Protein (TIRAP)/TRIF-related adaptor molecule (TRAM); this ultimately leads to an activation of the IκB kinase (IKK) complex (Fig. 3). The IKK complex consists of three subunits: the functionally non-redundant kinases IKKα and IKKβ and a regulatory subunit NEMO [27]. The signal transmission requires context-dependent non-degradative ubiquitinylation of RIPK1, NEMO and other intermediators, such as IL-1 receptor-associated kinase 1 (IRAK1) and TRAF6. IKKβ mediates the phosphorylation of IkBα at Ser32 and Ser36. The phosphorylated serines of the IκB members function as a binding site for WD-40 domains of F-box protein β-transducin repeat-containing proteins (β-TrCP), the substrate recognition subunits of the cullin-RING E3 ligase SCF^β−TrCP^ [29]. This process results in the Lys48-linked ubiquitinylation and the subsequent fast degradation of the IκBs, liberating NF-κB complexes (predominantly RelA:p50 and c-Rel:p50 dimers) to enter into the nucleus and switch on the transcription of target genes.

The non-canonical signaling pathway is activated in response to lymphotoxin (LT) α1β2 heterotrimers, receptor activator of NF-κB ligand (RANKL), the B cell activating factor (BAFF), CD40 ligand (CD40L), LIGHT, TNF-like weak inducer of apoptosis (TWEAK), but not to TNF and the most classical NF-κB stimuli [30]. The signals are transduced by specific TNF receptor family members, such as LTβ receptor (LTβR) and RANK, which are essential for lymph node and osteoclast genesis and homeostasis, and by the BAFF receptor, CD40 and CD27, which regulate B-cell survival and proliferation [26]. One important function of these stimuli is to prevent a degradation of the NF-κB inducing kinase (NIK), which further phosphorylates IKKα and recruits it into the p100 complex (associated with RelB) (Fig. 3). Ser866 and Ser870 phosphorylations are a prerequisite for a recruitment of the SCF^β−TrCP^ ubiquitin ligase, Lys48-linked ubiquitinylation at Lys855, and the subsequent processing of p100 by the 26S proteasome [31, 32]. Simplifying, the canonical pathway is implicated in the first line inflammatory response, and the non-canonical pathway is required for development of the lymphoid organs, e.g., lymph nodes, spleen and Peyer’s patches [33].

Both pathways are activated in *H. pylori*-infected cells, and this activation requires intact CagPAI [34–36]. The bacterial T4SS is strongly required for the NF-κB activation [37–39] (Fig. 2), but the exact mechanism is not clear. Intriguingly, an intermediate in bacterial LPS synthesis β-ADP heptose is crucial and induces a robust NF-κB response in AGS cells, when applied in vitro in concentrations low as 10–50 nM [28]. Whether live *H. pylori* delivers this compound to the host cell through the T4SS should be further clarified. The bacterial monosaccharides directly activate alpha-kinase 1 (ALPK1), which phosphorylates TRAF-interacting protein with forkhead‐associated (FHA) domain (TIFA). Activated TIFA oligomerizes and interacts eventually with signaling adaptor TRAF2 and, probably, with the RING-type E3 ubiquitin ligase TRAF6, which leads presumably to the TRAF6 activation [40, 41]. Activation of IKKs in an in vitro reconstitution system requires TIFA, TRAF6, the E2 enzyme Ubc13 (UBE2N), its cofactor Uev1A and the TGFβ-activated kinase 1 (TAK1) kinase complex. It confirms an important role of ubiquitinylation in the IKK activation [41]. The exact molecular mechanism of TRAF6 activation by (dimerized) TIFA is still a matter of investigation [42].

TRAF6 is known to catalyze the binding of Lys63-linked ubiquitin conjugates to NEMO, IRAK1, TAK1 and itself upon Toll-like receptor (TLR) and IL-1β receptor stimulation. Auto-ubiquitinylation promotes interaction of TRAF6 with UBDs of other proteins, creating a wide interaction network. In *H. pylori*-infected AGS cells, TRAF6 transiently associates with the molecular complex TAK1/TAB1/TAB2 most likely via the UBD of TAB2 [43]. TAK1 undergoes Ubc13-mediated Lys63-linked ubiquitinylation at Lys158 [44]. The complex further interacts with IKKs, leading to IκBα and RelA phosphorylation [45] (Fig. 3). Importantly, the TAK1/TAB1/TAB2 complex interacts with a number of proteins, including the RING-type E3 ligases tripartite motif (TRIM)21 and TRIM28, which could further modulate the downstream signaling cascade [45].

Fast degradation of IκBα after infection with *H. pylori* has been detected in several cell lines. The inhibition of proteasomal proteolysis with MG132 blocked both degradation of IκBα and IL-8 production induced by *H. pylori* [46]. Based on data from other experimental models, the SCF^β−TrCP^ E3 ubiquitin ligase is considered to be responsible for Lys48-linked ubiquitinylation of IκBα, despite of the fact that an involvement and the regulation of SCF^β−TrCP^ in *H. pylori* infection have never been addressed experimentally.

Two homologous F-box proteins, β-TrCP1 (FBXW1) and β-TrCP2 (FBXW11) are redundant in functions and, in addition to IκBα, p105 and p100, can target for proteasomal proteolysis some upstream components of the NF-κB signaling pathway, including IRAK1 and protein kinase D1 in response to IL-1 and LPS, respectively [47, 48]. Thus, these E3 ligases can both promote and inhibit NF-κB by targeting different effectors on different pathway levels. This can certainly complicate an interpretation of β-TrCP depletion experiments. In addition, many substrates of the SCF^β−TrCP^ are the hub regulators of cell cycle and proliferation (such as Wee1, Cdc25A, E3 ubiquitin ligase FBXO5 (Emi1), cyclin D1, β-catenin), migration (such as Snail, Twist), and apoptosis (BimEL, procaspase 3). It points to the complex role of the SCF^β−TrCP^ in cellular physiology and pathology [49]. The β‐TrCP gene is rarely mutated in gastric cancer: five somatic missense mutations in the β-TrCP coding gene were found by Kim et al. (2007) in about 5.2% (95 patients) of the investigated tissue samples. In all these samples, the stabilization of β-catenin, a classical substrate of the SCF^β−TrCP^, was detected by immunohistochemistry, despite genes encoding β‐catenin and its regulators Siah‐1, Axin, p53 and APC were not mutated [50].

The mechanism of the non-canonical NF-κB activation by *H. pylori* is not entirely resolved. The infection of epithelial cell lines with bacteria results in a T4SS-dependent induction of p100, its cleavage to p52, NIK stabilization, nuclear translocation of RelB:p52, and expression of chemokines CXCL13, CXCL10 and CCL20 [35, 36, 51]. It has been hypothesized that *H. pylori* either directly stimulated the LTβR during the T4SS—host cell plasma membrane interaction [36] or involved the canonical NF-κB, which induced expression of p100, LTα and LTβ genes [35]. In addition, *H. pylori* infection has been shown to stimulate expression of LIGHT (but not BAFF, CD40L and TWEAK) independently of canonical NF-κB in gastric cell cultures and biopsies from patients with gastritis and early gastric tumors [35]. However, it remains to be investigated, how NIK accumulation is achieved upon infection. In unstimulated cells, newly synthesized NIK rapidly binds to the adaptor protein TRAF3 and interacts thereby with the TRAF3-bound TRAF2 and E3 ligases cIAP1 and cIAP2. This leads to cIAP-mediated Lys48-linked ubiquitinylation of NIK and its proteasomal degradation [26, 30] (Fig. 3). The recruitment of some components of the complex to the TNF superfamily receptors in the non-canonical NF-κB pathway impedes TRAF3-TRAF2-cIAPs interaction and NIK ubiquitinylation. TRAF3 and TRAF2 can also be downregulated through Lys48-linked ubiquitinylation and cIAPs are also activated and de-activated through ubiquitinylation [52, 53]. In *H. pylori*-infected cells, it has been shown that TRAF2 becomes recruited to the LTβR [36].

The NF-κB pathways have been studied in conjunction with chronic inflammation and gastric cancer. Changes in the expression of p105/p50, RelA and IKKα, RelA phosphorylation and cellular localization, as well as overexpression of target genes, including IL-6, matrix metalloproteinases, VEGF, were described in gastric cancer tissue [19, 54]. An abnormal expression of TRAF6 detected in gastric cancer tissues can be causative for enhanced proliferation, impaired differentiation, promoted stemness and migration [55, 56]. Infection-induced or dysregulated NF-κB contributes to gastric carcinogenesis via supporting both sustained inflammation and survival of damaged cells [19].

### RING-type E3 ubiquitin ligases inhibitor of apoptosis (IAP)s at crossroads of inflammation and cell death

Members of the IAP group, also known as baculoviral IAP repeat containing (BIRC) proteins, act as E3 ubiquitin ligases towards initiator caspase 8 and effector caspases 3 and 7, RIPKs, TRAF2, NIK, ASK1, NEMO, IKKε, B-cell lymphoma/leukemia 10 (Bcl10), themselves, and their negative regulator DIABLO/second mitochondria-derived activator of caspases (SMAC). IAPs participate in signaling pathways triggered by the TNF receptor family, TLRs, NOD-like receptors and RIG-I-like receptors, and are involved in regulation of many cellular processes, including inflammatory signaling, proliferation, apoptosis and motility [57].

The human IAP family consists of NAIP (BIRC1), cIAP1 (BIRC2), cIAP2 (BIRC3), X-linked-IAP (XIAP, BIRC4), survivin (BIRC5), Bruce (BIRC6), Livin/melanoma‐IAP (ML-IAP, BIRC7), testis‐specific IAP (Ts‐IAP)/hILP2 (BIRC8). The IAPs contain 1–3 baculoviral IAP repeats (BIR) for protein–protein interaction. XIAP, cIAP1, cIAP2, ML-IAP, and ILP2 have an UBA domain, which enables the binding of polyubiquitin conjugates. All IAPs except survivin have also the RING and the caspase‐activating and recruitment domain (CARD), responsible for E3 ligase activity and protein–protein interactions, respectively [58, 59].

The interest in IAPs originates from their role in apoptosis. The extrinsic apoptotic pathway is activated following binding of FAS receptor (CD95) and TNF receptor with their ligands FasL and TNF, respectively. The intrinsic pathway is triggered by, e.g., toxins, hypoxia and DNA damage, and implements mitochondria. In both cases, caspases are sequentially activated, resulting in degradation of structure proteins and DNA fragmentation. The role of IAPs in regulation of apoptosis is mostly related to their ability to directly bind to and inhibit caspases 3, 7, 9 (described for XIAP) and to manipulate RIPK1 ubiquitinylation, which promotes pro-survival NF-κB and inhibits cell death in response to TNF (described for cIAP1 and cIAP2) [60].

In detail, an activation of the TNF receptor 1 leads to the assembly of the receptor-proximal complex-I consisting of TRADD/TRAF2/RIPK1/cIAP1 and cIAP2, where cIAPs became auto-ubiquitinylated and catalyze ubiquitinylation of RIPK1 and probably other interactors (Fig. 3). The non-degradative ubiquitinylations Met1, Lys11 and Lys63 assist in recruiting of the TAK1/TAB2/TAB3 and the E3 ligase linear ubiquitin chain assembly complex (LUBAC, composed of HOIL/HOIP/SHARPIN) to the receptor-proximal complex-I. Subsequently, LUBAC mediates linear ubiquitinylation of different components of complex-I (RIPK1, TRADD and TNF receptor 1) and supports recruitment and activation of IKKs [61]. It is believed that the described events prevent the formation of a pro-apoptotic signaling complex, referred to as complex-II or necrosome, in the TNF-initiated extrinsic apoptotic pathway [62]. Genetic deletion of cIAPs abrogates RIPK1 ubiquitinylation, leading to complex-II formation and RIPK1-mediated cell death in response to TNF [63].

On the other hand, cIAP1, via its UBA domain, can repress RIPK1 auto-activation and promote Lys48-linked ubiquitinylation and degradation of RIPK1. This could be a feedback mechanism to limit RIPK1-mediated proinflammatory or cytostatic effects [62]. In addition, cIAPs prevent constitutive activation of non-canonical NF-κB signaling through degradative ubiquitinylation of NIK, as described in the previous section (Fig. 3).

Dysregulation of cIAPs is implicated in the pathogenesis of several lymphoma types, including Burkitt lymphoma and mucosa-associated lymphoma tissue (MALT), lung cancer and X-Linked lymphoproliferative syndrome. Noteworthy, MALT lymphomas might be proceeded by chronic antigenic stimulation and are strongly associated with CagA( +) *H. pylori* infection [64, 65]. The most common structural chromosomal abnormality in MALT lymphoma is the generation of the *API2-MALT1* fusion transcript and chimeric protein, which comprises the N-terminal *API2*(cIAP2) region with three intact BIR domains and the C-terminal *MALT1* region containing an intact caspase-like domain. The cIAP2-MALT1 chimeric protein lacks E3 activity, and it leads to a decrease in ubiquitinylation and cleavage of cIAP2 and MALT1 target Bcl10 in MALT lymphomas. Bcl10 and cIAP2-MALT1 synergistically activate NF-κB and proliferation of lymphocytes [66]. In addition to its role in B- and T-cell signaling network, Bcl10 can function as an adapter protein downstream of the TNF receptor 1-TRADD-RIPK1 complex and NIK in other types of cells. MALT1 is involved in nuclear export of Bcl10 but can also interact and promote the activity of TRAF6. Worthy of note is that Bcl10 and MALT1 were not involved in the activation of NF-κB by *H. pylori* in epithelial AGS cells, as has been shown using specific siRNAs [67].

Increased expression of cIAP2 was found in 70% of human gastric cancer tissues if compared to non-cancerous gastric tissue, at both mRNA and protein levels (by PCR and immunohistochemistry (IHC)) [68]. cIAP2 (but not cIAP1 and XIAP) was found to be expressed at a higher level in well-differentiated gastric cancer cell lines (MKN7, MKN74 and NCI-N87) than in moderately (SGC-7901) or poorly differentiated gastric cancer cells (SNU-1, AGS), at the mRNA level. Depletion of cIAP2 in SGC-7901 cells resulted in a 30% decrease in cell proliferation, a 20% increase in apoptosis and delayed migration [68]. Some studies have found an enhanced expression of XIAP in the gastric carcinoma, which was associated with advanced stage and poor prognosis [69, 70].

Chang et al. (2004) have detected an increased protein level of cIAP in the cancer tissues compared to the adjacent non-cancer tissues, and in stomachs of *H pylori*-positive patients. Stage III and IV patients had significantly higher concentrations of IAP than stage I and II patients. Interestingly, similar data were obtained for NF-κB and iNOS, as well as for 8-OHdG concentration, which is an indicator for oxidative DNA damage [71]. Further, an increase of cIAP2 mRNA in subjects with *H. pylori*-positive atrophic gastritis and intestinal metaplasia or early gastric cancer was detected in comparison to *H. pylori*-negative controls without atrophic gastritis and intestinal metaplasia. In the *H. pylori* eradication group, expression of the cIAP2 mRNA and the protein significantly decreased and apoptosis increased at the 12-month follow-up after endoscopic submucosal dissection. Expressions of *survivin*, *cIAP1*, *XIAP*, and *NAIP* were not changed in this study [72]. Remarkably, in other studies it has been detected that the survivin protein level was decreased in the mucosa of patients with *H. pylori*-related gastritis [73]. In gastrointestinal cell culture, different *H. pylori* strains induced survivin downregulation, which correlated with apoptosis and loss of cell viability. Overexpression of survivin conferred the viability of the infected cells [73]. Further, an effect of a specific survivin inhibitor was studied in gastric cancer cell xenografts (without infection). Here, expansion and growth of the cancer stem cell-like cells was suppressed concomitantly with downregulation of the protein levels of β-catenin, c-Myc, cyclin D1 and CD44 [74].

With regard to cIAP2 in experimental infection models, Sydney Strain 1 of *H. pylori* led to an enhanced cIAP2 expression in gastric tissue of C57 mice already after 2 weeks post challenge [68]. In gastric epithelial cell lines MKN45 and AGS, CagPAI-positive *H. pylori* strains have been shown to up-regulate the expression of cIAP2 within the first hours post infection (p.i.) [75–77]. This effect was executed via NF-κB transcriptional activity, as it was demonstrated using NF-κB inhibitor CAPE [76]. An *H. pylori* infection is associated with a slightly induced apoptotic death in cell cultures [75, 77, 78], and depletion of cIAP2 (in MKN45 cells) potentiates this effect [76]. Therefore, *H. pylori*-activated NF-κB signaling pathway can support cell survival by up-regulating cIAP2 expression. Because of its central role in the non-canonical NF-κB pathway, cIAP2 prolonged upregulation could play an important role in *H. pylori*-related MALT lymphoma pathogenesis [64].

XIAP, which inhibits apoptosis through binding intrinsic caspases, can be activated by phosphorylation in a CagA-dependent manner, leading to enhanced ubiquitinylation and proteasomal degradation of the host proapoptotic factor Siva1, supporting thereby survival of human cells with damaged DNA [79]. It remains unknown, whether and how other IAPs (BIRC6-8) are regulated upon infection, despite the IAPs family members are expected to sensitize and de-sensitize host cells to inflammatory stress and apoptosis.

cIAPs can interact with the Wnt/β-catenin signaling pathway [80]. This link has not been investigated in the context of *H. pylori* infection yet, despite the Wnt/β-catenin has been shown to be induced by *H. pylori* [81, 82].

### E3 ubiquitin ligase MDM2, a central node in the p53 signaling pathway

Gastric carcinogenesis is strongly related to dysregulation of the cell cycle control. p53, a transcription factor and tumor suppressor, which regulates cell cycle arrest, DNA repair, apoptosis, senescence and autophagy, is mutated and deactivated in a set of gastric tumors [20, 83]. The protein is encoded by the *TP53* gene and can be represented by 12 isoforms (as a result of different splicing) with overlapping biologic activities and a role in cancer. The full-length (canonical) p53 protein contains a N-terminal transactivation domain (TAD, aa 1–42), four zinc finger loops (the DNA-binding domain, DBD, aa 100–300) essential for p53 conformation, and the C-terminal hinge and oligomerization domains. Through its N-terminal TAD and also the DBD, most of p53 isoforms interact with the dimeric E3 ligase murine double minute 2 protein (MDM2), which mediates their Lys48-linked ubiquitination and degradation by the proteasome.

Classically, p53 is stabilized in response to DNA damage, when ataxia telangiectasia mutated (ATM) and protein kinase DNA-activated (DNA-PK) phosphorylate Ser15 and Ser37 of p53, thereby preventing MDM2-p53 interaction. An increase in the protein level and in the nuclear activity of p53 leads to an arrest of the cell cycle to allow cell repair and to prevent propagation of affected cells. Other mechanisms leading to p53 stabilization include disabling MDM2 activity through PTMs or binding with the ARF group of proteins (p19ARF, for example) [84] (Fig. 3). It has been suggested that ubiquitinylation of the p53 isoforms may also be associated with proteasome-independent functions, including the regulation of subcellular location and protein interaction [85].

In addition to its major role in p53 inhibition, MDM2 seems to participate in the downregulation of DNA replication or repair independently of p53, e.g., via binding to repair proteins, NBS1 and DNA polymerase ϵ [86]. When *MDM2* is overexpressed, damaged cells can escape from the cell cycle checkpoint control and become carcinogenic.

It is known that p53 can induce MDM2; MDM2 then downregulates p53 through a negative feedback loop [87]. Perhaps, in long-term infection with *H. pylori*, especially with virulent CagPAI-positive strains, this regulatory network becomes more complex, ending finally in dysfunction of the MDM2-p53 axis in parallel to genomic instability in gastric tissue [88]. The IHC analysis of human gastric mucosa revealed p53 overexpression in chronic gastritis [89]. The MDM2 level has been found to progressively increase in gastric specimens from normal gastric mucosa, chronic gastritis, intestinal metaplasia, dysplasia, and gastric cancer. In stomachs of *H. pylori*-infected individuals, MDM2 and p53 were enhanced and successful *H. pylori* eradication downregulated them [90]. An association of *H. pylori* infection with the MDM2 and p53 amplifications was found in dysplasia and metaplastic atrophy, respectively [91, 92]. Further, the progression to gastric cancer frequently involves mutations in *TP53* and loss of its function [20].

It has been demonstrated in *H. pylori*-infected Mongolian gerbils, that p53 was strongly up-regulated in the antrum at 4–8 h p.i. At 24 h, the levels of p53 decreased despite the presence of bacteria and stayed low up to 2 weeks p.i. The second peak of p53 up-regulation accompanied by intense inflammation was detected 12 weeks p.i. [93]. Similarly, the levels of p53 rapidly increased and then dropped down following a co-culture of SNU1 and AGS gastric epithelial cells with *H. pylori* strains 7.13 or J166. The decline in p53 level was regulated post-translationally by MDM2 (HDM2), which was not increased in amount but phosphorylated at Ser166 by Akt and Erk kinases in infected cells [93, 94]. The activity of the E3 ubiquitin ligase and the p53 status in *H. pylori*-infected cells were additionally controlled by its interactor and tumor suppressor p14ARF (CDKN2A), which is known to block MDM2 nucleocytoplasmic shuttling and activity [95]. It has been shown that *H. pylori* induces p14ARF ubiquitinylation (and degradation) by the HECT domain-containing E3 ligase TRIP12 (Fig. 3). An increased expression of TRIP12 protein was found in infected gastric epithelial cell culture and human gastric mucosa of *H. pylori*-infected patients [96]. Interestingly, the authors have suggested that *H. pylori*’s CagA promoted p14ARF degradation, MDM2 (HDM2) phosphorylation and degradation of p53. Another group published that the treatment of GES-1 cells with *H. pylori* filtrates induced expressions of the phosphorylated Akt kinase and MDM2 accompanied by cytostatic and cytotoxic effects. Here, the p53 level was found to be increased, perhaps because the DNA-damage-induced upregulation of the p53 expression was not balanced by *H. pylori*-induced MDM2-mediated p53 degradation [92]. Ye et al. found that LPS from *H. pylori* strain 26695 downregulated the expression of miR-375 leading to SP1 activation and increased expression of MDM2 in gastric epithelial cells [97]. Thus, these experimental models provide ambiguous results concerning bacterial effectors and mechanisms in MDM2-p53 pathway regulation. Certainly, further in vitro and in vivo studies in this field are required.

Genotyping as well as meta-analyses determined that the p53-responsive intronic promoter region of the *MDM2* gene contains a functional single-nucleotide T–G polymorphism, known as SNP309. The G allele may increase the binding affinity for the transcription factor Sp1, leading to elevated MDM2 expression [98]. A significant association between individuals with the GG genotype or TG genotype and an elevated risk of stomach and some other types of cancer, when compared to subjects with the TT genotype, was demonstrated [98–100]. In different human populations, an association of increased risk of gastric cancer with the GG genotype of *MDM2* was especially evident among *H. pylori*-infected patients [99, 101–103].

### A20 (TNFAIP3, OTUD7C) in inflammation and cell survival

A20 is a component of the A20 ubiquitin-editing complex comprising of RNF11, the HECT-type E3 ubiquitin ligase ITCH and Tax1 binding protein 1 (TAX1BP1) [104]. The N-terminal OTU domain of A20 is responsible for dimerization; it contains the catalytic cysteine 103 and confers hydrolase activity of the enzyme towards activating Lys63-linked ubiquitin chains. The C-terminal seven zinc finger domains are required for binding to Lys63- and Met1-linked ubiquitin chains on A20 binding partners, including RIPK1, NEMO, TRAF2 and TRAF6 (Fig. 3). This binding promotes Lys48-linked ubiquitinylation and the degradation of at least RIPK1, which terminates TNF- and LPS-triggered signaling [105, 106]. Additionally to the TRAF6 deubiquitinylation, A20 disrupts the interactions between TRAF6 and the E2 ubiquitin conjugating enzymes Ubc13 and UbcH5 (UBE2D1), and supports Lys48-linked ubiquitinylation and proteasomal degradation of both Ubcs [107], affecting further IL-1β- and PAMPs-triggered signal transmission.

A20 deubiquitinylates MALT1 and NEMO. IKKβ phosphorylates A20 at Ser381, which increases the ability of A20 to inhibit the NF-κB signaling pathway [108]. IKKβ phosphorylates TAX1BP1 on Ser593 and Ser624, thereby activating the A20 ubiquitin-editing complex in response to TNF or IL-1β [104]. As a result of the NF-κB stimulation by TNF, IL-1β or PAMPs, A20 is overexpressed and functions as a negative regulator of the signaling pathway (Fig. 3). Reduced expression of A20 in follow of single nucleotide polymorphisms (SNPs) in the A20 gene is linked to inflammatory disorders, including rheumatoid arthritis, systemic lupus erythematosus, psoriasis and inflammatory bowel disease. Somatic mutations in A20 could contribute as drivers in oncogenesis of *H. pylori*-eradication-resistant gastric marginal zone lymphoma [109].

In case of *H. pylori*, infection of MKN45 or AGS cells with cagPAI-positive *H. pylori* but not with isogenic CagE-deficient (thus, T4SS-invalid) mutant, significantly up-regulated the mRNA and the protein amount of A20 [110]. The overexpression of A20 resulted in an inhibition of *H. pylori*-mediated NF-κB activation thus confirming the negative feedback regulatory mechanism. In parallel to the restriction of pro-survival NF-κB, A20 DUB activity counteracts cullin-3-mediated Lys63-linked ubiquitinylation of procaspase 8, thereby inhibiting caspase 8 and apoptotic cell death in gastric epithelial cells. This mechanism can support the bacterial colonization of gastric epithelia to establish long-term infection [77]. Somewhat contraposing data were obtained by Sun et al. [111]. There, A20 was characterized as a direct target of miR-29a-3p up-regulated in BGC-823 and GES-1 cells following infection with the *H. pylori* strain 26695. The authors found a decreased expression of A20 in these cells as well as in *H. pylori*-positive gastric mucosa tissue compared with *H. pylori*-negative gastric mucosa by IHC. It has been suggested that the miR-29a-3p-related A20 reduction was responsible for the up-regulation of Snail, vimentin and N-cadherin expressions, and for an enhanced migration of human gastric epithelial cells [111].

### Pellino

Pellinos 1, 2 and 3 are known mostly for their role in the regulation of TLR- and IL-1β receptor-triggered signal transmission. They possess an N‐terminal FHA domain that mediates association with IRAKs, and a C‐terminal RING-type domain that confers the E3 ubiquitin ligase activity [112]. Pellinos interact with IRAK1, IRAK4, MyD88 and TRAF6 and mediate Lys63-linked ubiquitinylation of IRAK1, promoting thereby recruitment of the TAK1/TAB1/TAB2 complex into signaling pathway (Fig. 3). TAK1 further activates IKKs or MAP kinases leading to an activation of NF-κB, AP-1 and ELK1-dependent gene transcription [113]. Pellino1 can promote Lys63-linked ubiquitinylation of RIPK1 and Lys48-linked ubiquitinylation of RIPK3 (in relation to negative regulation of necroptosis) [114]. Smith et al. [115] have investigated a role of the Pellino proteins in *H. pylori* infection. They used the human embryonic kidney cells stably expressing TLR2. TLR2 is known to recognize bacterial lipoteichoic acid, di- and tri-acylated cysteine-containing lipopeptides. In HEK-TLR2 cells, Pellino1 and Pellino2 contributed to NF-κB activation (in the transactivation assay) and to IL-8 mRNA synthesis in response to *H. pylori* LPS. On the other hand, Pellino3 exerted a negative modulatory role. Pellino1 was significantly higher expressed in gastric epithelial cells and gastric tissue than Pellino3, and *H. pylori* LPS further amplified the Pellino1 expression in gastric epithelial MKN45 cells [115]. Thus, Pellino1 is up-regulated following the infection and further supports NF-κB signaling pathway and host inflammatory response.

### SCF substrate-recognition subunit FBXW7

F-box/WD repeat domain containing 7 (FBXB7), similar to β-TrCP, is a substrate-recognition component in a SCF E3 ubiquitin ligase complex, with specificity towards so called Cdc4 phospho-degron (CPD). It catalyzes Lys48-linked ubiquitinylation of a number of proteins, including cyclins E and D1, transcriptional regulator and proto-oncogene c-Myc, type I transmembrane receptor NOTCH1, kinase mammalian target of rapamycin (mTOR) and p100 (NF-kB2) [116–118] (Fig. 3). The SCF^FBXW7^ negatively regulates JNK signaling pathway via degradative ubiquitinylation of the downstream effectors JUN phosphorylated by glycogen synthase kinase 3β (GSK3β) [119]. Thus, FBXW7 is known for its role in regulation of cell cycle, cell proliferation and differentiation [120].

In mammals, three isoforms are described: FBXW7α (located in the nucleoplasm), FBXW7β (in the endoplasmic reticulum) and FBXW7γ (in the nucleolus), with FBXW7α being the most abundant in proliferating cells [120, 121]. The isoforms contain a dimerization domain, F-box domain (bins to Skp1 adaptor protein within the SCF) and seven tandem WD-40 repeats (recognize phosphorylated substrate), and differ in their N-terminal region [121]. The isoforms seem to be regulated by different promoters and transcriptional factors (expression of FBXW7β but not of FBXW7α is p53-dependent, for example) and have specific functions [122].

In gastric carcinoma, a loss of heterozygosity of FBXW7 occurred in 32% of early-onset gastric cancers and correlated with a loss of FBXW7 expression in 26%, together with an upregulation of c-Myc (by IHC) [117]. Deletions of one copy of FBXW7 and p53 were observed in 45.5% and 21.2% of gastric tumors, respectively, and their mRNA levels were decreased in tumors [123]. Deregulation of FBXW7 and amplification of c-Myc were associated with the presence of lymph node metastasis and tumor stage III-IV [123]. In gastric adenocarcinoma cell lines ACP02 and ACP03, increased c-Myc and reduced FBXW7 expression were associated with a more invasive phenotype [123].

FBXW7 can be also regulated by several microRNAs. miR-25, whose increased expression in primary gastric tumors inversely correlated with amount of FBXW7, was related to a more aggressive cancer phenotype. The challenge with miR-25 construct or FBXW7 siRNA promoted proliferation, invasion and migration of HGC-27 and SGC-7901 gastric cancer cells [124]. miR-223, which also directly targets FBXW7, has been found to be significantly up-regulated in cisplatin-resistant gastric cancer cells SGC-7901 and BGC-823 as well as in *H. pylori*-infected gastric cancer tissue [125]. Downregulation of miR-223 and overexpression of FBXW7 affected the G1/S transition of cell cycle by downregulating, e.g., CDK2, CDK4, CDK6, cyclins D1, D2 and D3 [125] (Fig. 3). Similarly, the suppression of miR-223 restored the FBXW7 expression and the sensitivity of HER2-positive gastric cancer cells to trastuzumab through the modulation of apoptosis [126]. Thus, the tumor suppressor FBXW7 affects the proliferation/survival of gastric cancer cells, and can be regulated following infection and chemotherapeutic treatment.

### RNF43 in β-catenin signaling pathway

RNF43 and its functional homolog zinc and RING finger 3 (ZNRF3) belong to the Goliath and Godzilla families of transmembrane RING-type E3 ligases. They have an N-terminal extracellular region, a transmembrane domain and an intracellular C-terminal RING-type domain, thus, represent a type I transmembrane receptor [127, 128]. These E3 ubiquitin ligases act as negative feedback regulators of the Wnt/β-catenin signal transmission by promoting ubiquitinylation, endocytosis and subsequent degradation of Wnt receptor Frizzled and co-receptor LRP6 [129, 130]. In the absence of Wnt, GSK3β phosphorylates β-catenin and initiates thereby its Lys48-linked ubiquitinylation by SCF^β−TRCP^. The activation of Wnt receptor inhibits GSK3β activity through Dishevelled (Dsh), resulting in dephosphorylation and stabilization of the β-catenin (Fig. 3). Further, β-catenin accumulates in the nucleus and co-activates the T-cell factor/lymphoid enhancer factor (TCF/LEF) transcription factors, leading to expression of cyclin D1 and c-Myc [130] (Fig. 3). Upon infection with *H. pylori* and in gastric cancer cells, β-catenin is often up-regulated in follow of mutations, changes in GSK3β upstream regulation, or silenced gene expression of E-cadherin, a β-catenin binding partner in cellular adherens junctions [17, 131] (Fig. 3).

RNF43 mutations have been found to accompany the transition from adenoma to dysplasia and gastric cancer [132, 133]. The loss of RNF43 is associated with distant metastasis and a poor prognosis for gastric cancer patients [134]. Experimental overexpression of RNF43 in gastric tumor-derived cancer stem cells downregulates the β-catenin, TCF4 and c-Myc protein levels, sensitizes cells to chemotherapy and impairs their tumorigenicity in vivo [134].

RNF43^H292R/H295R^ mice bearing transactivating mutations in the RING domain are more sensitive to *H. pylori* infection towards the development of gastritis and lymphocyte infiltration, compared to the wild-type mice. Furthermore, infected mutant mice developed atrophy, hyperplasia and mucin 2 expressing metaplasia [135]. In AGS and MKN45 cells, RNF43 depletion mitigated the DNA damage response and apoptosis induced by *H. pylori*-infection, γ-radiation, 5-fluorouracil and cisplatin [136]. Further investigations concerning the RNF43 substrates are required to show specificity of this E3 ubiquitin ligase and, thus, to explore its therapeutic potential.

### RNF180

It is not much known about the function of the RING-type E3 ligase RNF180. This recently discovered ligase is predicted to be an integral membrane protein located mainly on the cytoplasmic side of the endoplasmic reticulum. It associates with the E2 ubiquitin‐conjugating enzyme UbcH6 and promotes proteolysis of a substrate in vitro. In addition, RNF180 itself is a substrate for ubiquitinylation and proteasomal degradation [137].

Promoter methylation of RNF180 was detected in 76% (150 of 198) of primary gastric cancers and in 55% (11 of 20) of intestinal metaplasia but in none of 23 normal gastric tissues [138]. The RNF180 mRNA levels in gastric cancer samples were relatively low [139]. The presence of *H. pylori* was associated with an increased RNF180 promoter methylation in normal tissue or by mild gastritis and an increased hypermethylation in atrophic gastritis [139]. RNF180 was not expressed in AGS, Kato III, MKN28, N87 and SNU1 gastric cancer cell lines. Its re-expression suppressed cell growth and induced apoptosis by up-regulating the anti-proliferative Metastasis Suppressor Protein 1 (MTSS1), p14ARF and the pro-apoptotic tissue inhibitor of metalloproteinases 3 (TIMP3) [138] (Fig. 3).

### Seven in absentia homologue (Siah) 2

The RING-type E3 ligases Siah1 and Siah2 have overlapping functions—they either directly ubiquitinylate their substrates, including TRAF2, or assist in multimolecular ubiquitinylation complexes, such as the β-catenin complex. The activity of Siah2 towards TRAF2 suggests its suppressive role in TNF-initiated NF-κB regulation [140]. The Siah E3 ligases are also known for their role in targeting proline hydroxylases PHD1 and PHD3 for proteasomal degradation (Fig. 3). Under normoxic conditions, the PHDs hydoxylate hypoxia-inducible factor-α (HIFα) transcription factors. Further, HIFα undergo a degradative ubiquitinylation by the CRL2^VHL^. Hypoxia stimulates transcription of *Siah* (through the HIFα/β heterodimer binding to hypoxia response elements at the gene loci), which results in inhibition of PHDs and stabilization of HIFs [141]. The accumulated HIFs up-regulate tumorigenic genes, including *VEGFA*. Increased Siah levels and its nuclear accumulation are associated with progression of breast, prostate and liver cancers [142].

In the gastric cancer cells AGS, Kato III and MKN45, *H. pylori* infection induced an expression of Siah2, in a E26 transformation-specific sequence 2 (ETS2)- and Twist-related protein 1 (Twist1)-dependent manner, which was accompanied by increased invasiveness and migration. Induced expression of ETS2, Twist1 and Siah2 was found in gastric cancer biopsies compared with noncancerous gastric tissue [143]. Further, Siah2 is stabilized via p300-driven acetylation at the lysine 139 residue in infected cells, which might increase its ligase activity towards PHD3 and cause HIFα accumulation in gastric epithelium. The increased acetylation of Siah2, absence of PHD3 and accumulation of HIFα were detected in the *H. pylori*-infected human gastric metastatic cancer biopsies and in invasive murine gastric cancer tissues [144].

### HACE1

The HECT domain-containing E3 ligase HACE1 has been described to target GTP-bound Rac Family Small GTPase 1 (Rac1) for ubiquitinylation and subsequent degradation, thereby regulating cell motility and host defense against pathogens [145]. HACE1 is involved in the Golgi biogenesis by regulating Rab proteins and the Golgi membrane dynamics [146]. HACE1 can participate in the autophagy, mitophagy and oxidative damage response [147]. There are few existing data regarding the HACE1 regulation. However, it has been found that HACE1 is downregulated in different human malignancies, including Wilms tumor and neuroblastoma. HACE1 loss promotes tumor growth, invasion, and metastasis; therefore, this E3 ubiquitin ligase functions as a tumor suppressor [148]. The reduction in HACE1 expression is caused by gene promoter hypermethylation, also in a set of primary gastric carcinomas and in AGS and MKN1 cells [149, 150]. Yoon et al. [150] have found *Hace1* hypermethylation in the gastric mucosa with *H*. *pylori* infection, atrophy and intestinal metaplasia, which was closely associated with an increased transcription of DNA methyltransferase 1 gene [150]. An overexpression of HACE1 in AGS and CGS7901 cells downregulated the protein level of β-catenin and inhibited the activity of the Wnt/β-catenin signaling pathway [151].

## Ubiquitinylation targeting in gastric cancer. Concluding remarks

The activation/deactivation of molecular signaling pathways, including the NF-κB, p53 and Wnt/β-catenin, in *H. pylori* infection and gastric cancer involves changes in the function, regulation and expression of E3 ubiquitin ligases and other components of the ubiquitinylation machinery. Some of these changes determine disease progression and could be explored for therapeutic targeting [152].

In experimental models of gastric cancer, the approved proteasome inhibitor bortezomib, the nonapproved indirect inhibitor of cullin-RING E3 ligases MLN4924 (pevonedistat) and small molecule inhibitors of the E3 ligase MDM2 nutlin-3 and APG-115 have demonstrated anti-proliferative effects and could prospectively supplement conventional chemo- and radiotherapy [153–155]. Currently, bortezomib is included in treatment protocols for multiple myeloma, diffuse large B-cell lymphoma, colorectal cancer and thyroid carcinoma; pevonedistat, APG-115 and derivatives of nutlin-3 are in preclinical and clinical trials involving patients with hematological malignancies and solid tumors but not patients with gastric cancer [156]. Mechanistically, nutlins and APG-115 increase the level of p53 and thus should be effective in a set of tumors harboring wild-type p53.

A number of inhibitors of ubiquitinylation have been identified to target the NF-κB signaling pathway; however, they have not been explored in relation to gastric cancer. For example, NSC697923, an inhibitor of the E2 enzyme Ubc13, has been shown to inhibit the formation of Lys63-linked ubiquitin chains, NF-κB activity and the proliferation of diffuse large B-cell lymphoma cells; it is currently in preclinical trials in the context of melanoma, B-cell lymphoma, neuroblastoma and colorectal cancer [156].

The activity of E3 ligases can be controlled indirectly by manipulating their regulatory proteins. For example, several mimetics of SMAC, an endogenous inhibitor of cIAPs, were developed and have progressed to clinical trials [157]. Compounds such as birinapant, LCL161 and Debio 1143 (AT-406) exhibit cytotoxic effects in head and neck squamous cell carcinoma cells with genomic amplification of Fas-associated Death Domain (FADD), cIAP1 or cIAP2 and in inflammatory breast cancer cells overexpressing XIAP, as well as in human hepatocellular carcinoma cell lines or in MDA-MB-231 breast cancer xenograft models [158]. The explored SMAC mimetics were ineffective as monotherapies in human studies; however, their clinical potential in combination approaches is currently under investigation.

Inhibition of pro-oncogenic microRNAs, e.g., the abovementioned miR-25 and miR-223 targeting FBXW7, is a potential strategy to control the activity of ubiquitin ligases. Transfection with targeting oligonucleotides to downregulate miR-223 increased the amount of FBXW7 and the sensitivity of gastric cancer cells to cisplatin and trastuzumab in culture [125, 126]. The natural compounds genistein and epigallocatechin-3-gallate repressed miR-223 in pancreatic cancer cells and miR-25 in breast cancer MCF-7 cells, respectively, finally enhancing apoptosis [159, 160]. Although considered oncomarkers for brain, lung, breast, prostate, thyroid and gastric cancers, these miRs target a broad range of substrates for normal cellular homeostasis. Thus, their inhibition can be harmful in untransformed cells or result in unwanted side effects in cancer cells [161].

Recent advances in the ubiquitinylation field have allowed the development of cell-permeable molecules called Proteolysis Targeting Chimeras (PROTACs) for selective targeting of different proteins by linking them to an E3 ubiquitin ligase for degradative ubiquitination [162, 163]. A PROTAC for MDM2, for example, consists of a nutlin-based MDM2 ligand joined to the E3 ubiquitin ligase cereblon (CRBN; CRL4^CRBN^) via a short linker and promotes efficient degradation of MDM2 in leukemia cells [164, 165]. The E3 ubiquitin ligases SCF^β−TRCP^, CRL2^VHL^, IAPs and MDM2 are tools in this promising technology to target proteins, which confer inflammatory effects, tumorigenesis and radiotherapy resistance (such as histone deacetylases, androgen and estrogen receptors, EGFR, and CDKs), for ubiquitin-mediated degradation [163]. This new protein engineering approach is able to guide the development of advanced therapeutics for different pathologies, including gastric cancer.

In conclusion, there is an increasing appreciation for the development of therapeutics suitable for targeting individual E3 ligases. However, further intense research on the catalytic sites, substrate-induced conformational changes, differences in ubiquitin binding, and intracellular regulatory networks is necessary to elucidate E3 ubiquitin ligases as specific targets or tools in gastric cancer therapy.

## References

[1] Rape M (2018). Ubiquitylation at the crossroads of development and disease. Nat Rev Mol Cell Biol.

[2] Streich FC (2014). Lima CD structural and functional insights to ubiquitin-like protein conjugation. Annu Rev Biophys.

[3] Cotton TR, Lechtenberg BC (2020). Chain reactions: molecular mechanisms of RBR ubiquitin ligases. Biochem Soc Trans.

[4] Morreale FE, Walden H (2016). Types of ubiquitin ligases. Cell.

[5] Dittmar G, Winklhofer KF (2019). Linear ubiquitin chains: cellular functions and strategies for detection and quantification. Front Chem.

[6] Vere G, Kealy R, Kessler BM, Pinto-Fernandez A (2020). Ubiquitomics: an overview and future. Biomolecules.

[7] Harrigan JA, Xavier Jacq X, Martin NM, Stephen P, Jackson SP (2018). Deubiquitylating enzymes and drug discovery: emerging opportunities. Nat Rev Drug Discov.

[8] Ribet D, Cossart P (2018). Ubiquitin, SUMO, and NEDD8: key targets of bacterial pathogens. Trends Cell Biol.

[9] Ashida H, Kim M, Schmidt-Supprian M, Ma A, Ogawa M, Sasakawa C (2010). A bacterial E3 ubiquitin ligase IpaH9.8 targets NEMO/IKKgamma to dampen the host NF-kappaB-mediated inflammatory response. Nat Cell Biol.

[10] Lin AE, Guttman JA (2012). The Escherichia coli adherence factor plasmid of enteropathogenic Escherichia coli causes a global decrease in ubiquitylated host cell proteins by decreasing ubiquitin E1 enzyme expression through host aspartyl proteases. Int J Biochem Cell Biol.

[11] Sanada T, Kim M, Mimuro H, Suzuki M, Ogawa M, Oyama A, Ashida H, Kobayashi T, Koyama T, Nagai S, Shibata Y, Gohda J, Inoue J, Mizushima T, Sasakawa C (2012). The Shigella flexneri effector OspI deamidates UBC13 to dampen the inflammatory response. Nature.

[12] Bray F, Ferlay J, Soerjomataram I, Siegel RL, Torre LA, Jemal A (2018). Global cancer statistics 2018: GLOBOCAN estimates of incidence and mortality worldwide for 36 cancers in 185 countries. CA Cancer Clin J.

[13] Tegtmeyer N, Wessler S, Necchi V, Rohde M, Harrer A, Rau TT, Asche CI, Boehm M, Loessner H, Figueiredo C, Naumann M, Palmisano R, Solcia E, Ricci V, Backert S (2017). *Helicobacter pylori* employs a unique basolateral type IV secretion mechanism for CagA delivery. Cell Host Microbe.

[14] Javed S, Skoog EC, Solnick JV (2019). Impact of *Helicobacter pylori* virulence factors on the host immune response and gastric pathology. Curr Top Microbiol Immunol.

[15] Nishikawa H, Hatakeyama M (2017). Sequence polymorphism and intrinsic structural disorder as related to pathobiological performance of the *Helicobacter pylori* CagA oncoprotein. Toxins (Basel).

[16] Sokolova O, Vieth M, Naumann M (2013). Protein kinase C isozymes regulate matrix metalloproteinase-1 expression and cell invasion in *Helicobacter pylori* infection. Gut.

[17] Naumann M, Sokolova O, Tegtmeyer N, Backert S (2017). *Helicobacter pylori*: a paradigm pathogen for subverting host cell signal transmission. Trends Microbiol.

[18] Williams LM, Gilmore TD (2020). Looking down on NF-κB. Mol Cell Biol.

[19] Sokolova O, Naumann M (2017). NF-κB signaling in gastric cancer. Toxins (Basel).

[20] Sokolova O, Naumann M (2019). Crosstalk between DNA damage and inflammation in the multiple steps of gastric carcinogenesis. Curr Top Microbiol Immunol.

[21] Coombs N, Sompallae R, Olbermann P, Gastaldello S, Göppel D, Masucci MG, Josenhans C (2011). *Helicobacter pylori* affects the cellular deubiquitinase USP7 and ubiquitin-regulated components TRAF6 and the tumour suppressor p53. Int J Med Microbiol.

[22] Álvarez A, Uribe F, Canales J, Romero C, Soza A, Peña MA, Antonelli M, Almarza O, Cerda O, Toledo H (2017). KCTD5 and ubiquitin proteasome signaling are required for *Helicobacter pylori* adherence. Front Cell Infect Microbiol.

[23] Necchi V, Sommi P, Ricci V, Solcia E (2010). In vivo accumulation of *Helicobacter pylori* products, NOD1, ubiquitinated proteins and proteasome in a novel cytoplasmic structure. PLoS ONE.

[24] Taniguchi K, Karin M (2018). NF-κB, inflammation, immunity and cancer: coming of age. Nat Rev Immunol.

[25] Neumann M, Naumann M (2007). Beyond IkappaBs: alternative regulation of NF-kappaB activity. FASEB J.

[26] Sun SC (2017). The non-canonical NF-kappaB pathway in immunity and inflammation. Nat Rev Immunol.

[27] Hayden MS, Ghosh S (2012). NF-kappaB, the first quarter-century: remarkable progress and outstanding questions. Genes Dev.

[28] Pfannkuch L, Hurwitz R, Traulsen J, Sigulla J, Poeschke M, Matzner L, Kosma P, Schmid M, Meyer TF (2019). ADP heptose, a novel pathogen-associated molecular pattern identified in Helicobacter pylori. FASEB J.

[29] Perkins ND (2007). Integrating cell-signalling pathways with NF-kappaB and IKK functionNat Rev. Mol Cell Biol.

[30] Yang XD, Sun SC (2015). Targeting signaling factors for degradation, an emerging mechanism for TRAF functions. Immunol Rev.

[31] Amir RE, Haecker H, Karin M, Ciechanover A (2004). Mechanism of processing of the NF-kappa B2 p100 precursor: identification of the specific polyubiquitin chain-anchoring lysine residue and analysis of the role of NEDD8-modification on the SCF(beta-TrCP) ubiquitin ligase. Oncogene.

[32] Liang C, Zhang M, Sun SC (2006). beta-TrCP binding and processing of NF-kappaB2/p100 involve its phosphorylation at serines 866 and 870. Cell Signal.

[33] Krappmann D, Vincendeau M (2016). Mechanisms of NF-kappaB deregulation in lymphoid malignancies. Semin Cancer Biol.

[34] Backert S, Naumann M (2010). What a disorder: proinflammatory signaling pathways induced by Helicobacter pylori. Trends Microbiol.

[35] Mejías-Luque R, Zöller J, Anderl F, Loew-Gil E, Vieth M, Adler T, Engler DB, Urban S, Browning JL, Müller A, Gerhard M, Heikenwalder M (2017). Lymphotoxin β receptor signalling executes Helicobacter pylori-driven gastric inflammation in a T4SS-dependent manner. Gut.

[36] Feige MH, Vieth M, Sokolova O, Täger C, Naumann M (1865). *Helicobacter pylori* induces direct activation of the lymphotoxin beta receptor and non-canonical nuclear factor-kappa B signaling. Biochim Biophys Acta Mol Cell Res.

[37] Schweitzer K, Sokolova O, Bozko PM, Naumann M (2010). *Helicobacter pylori* induces NF-kappaB independent of CagA. EMBO Rep.

[38] Sokolova O, Borgmann M, Rieke C, Schweitzer K, Rothkötter HJ, Naumann M (2013). *Helicobacter pylori* induces type 4 secretion system-dependent, but CagA-independent activation of IkappaBs and NF-kappaB/RelA at early time points. Int J Med Microbiol.

[39] Gall A, Gaudet RG, Gray-Owen SD, Salama NR (2017). TIFA signaling in gastric epithelial cells initiates the cag type 4 secretion system-dependent innate immune response to helicobacter pylori infection. MBio.

[40] Zimmermann S, Pfannkuch L, Al-Zeer MA, Bartfeld S, Koch M, Liu J, Rechner C, Soerensen M, Sokolova O, Zamyatina A, Kosma P, Mäurer AP, Glowinski F, Pleissner KP, Schmid M, Brinkmann V, Karlas A, Naumann M, Rother M, Machuy N, Meyer TF (2017). ALPK1- and TIFA-dependent innate immune response triggered by the helicobacter pylori type IV secretion system. Cell Rep.

[41] Ea CK, Sun L, Inoue J, Chen ZJ (2004). TIFA activates IkappaB kinase (IKK) by promoting oligomerization and ubiquitination of TRAF6. Proc Natl Acad Sci USA.

[42] Nakamura T, Hashikawa C, Okabe K, Yokote Y, Chirifu M, Toma-Fukai S, Nakamura N, Matsuo M, Kamikariya M, Okamoto Y, Gohda J, Akiyama T, Semba K, Ikemizu S, Otsuka M, Inoue JI, Yamagata Y (2020). Structural analysis of TIFA: Insight into TIFA-dependent signal transduction in innate immunity. Sci Rep.

[43] Sokolova O, Kähne T, Bryan K, Naumann M (2018). Interactome analysis of transforming growth factor-β-activated kinase 1 in Helicobacter pylori-infected cells revealed novel regulators tripartite motif 28 and CDC37. Oncotarget.

[44] Lamb A, Chen J, Blanke SR, Chen LF (2013). *Helicobacter pylori* activates NF-κB by inducing Ubc13-mediated ubiquitination of lysine 158 of TAK1. J Cell Biochem.

[45] Sokolova O, Maubach G, Naumann M (1843). MEKK3 and TAK1 synergize to activate IKK complex in Helicobacter pylori infection. Biochim Biophys Acta.

[46] Nozawa Y, Nishihara K, Peek RM, Nakano M, Uji T, Ajioka H, Matsuura N, Miyake H (2002). Identification of a signaling cascade for interleukin-8 production by *Helicobacter pylori* in human gastric epithelial cells. Biochem Pharmacol.

[47] Cui W, Xiao N, Xiao H, Zhou H, Yu M, Gu J, Li X (2012). β-TrCP-mediated IRAK1 degradation releases TAK1-TRAF6 from the membrane to the cytosol for TAK1-dependent NF-κB activation. Mol Cell Biol.

[48] Liu J, Yuan Y, Xu J, Xiao K, Xu Y, Guo T, Zhang L, Wang J, Zheng H (2018). beta-TrCP restricts lipopolysaccharide (LPS)-induced activation of TRAF6-IKK pathway upstream of IkappaBalpha Signaling. Front Immunol.

[49] Zheng N, Zhou Q, Wang Z, Wei W (1866). Recent advances in SCF ubiquitin ligase complex: clinical implications. Biochim Biophys Acta.

[50] Kim CJ, Song JH, Cho YG, Kim YS, Kim SY, Nam SW, Yoo NJ, Lee JY, Park WS (2007). Somatic mutations of the beta-TrCP gene in gastric cancer. APMIS.

[51] Maubach G, Sokolova O, Täger C, Naumann M (2020). CEACAMs interaction with *Helicobacter pylori* HopQ supports the type 4 secretion system-dependent activation of non-canonical NF-kappaB. Int J Med Microbiol.

[52] Zarnegar BJ, Wang Y, Mahoney DJ, Dempsey PW, Cheung HH, He J, Shiba T, Yang X, Yeh WC, Mak TW, Korneluk RG, Cheng G (2008). Noncanonical NF-kappaB activation requires coordinated assembly of a regulatory complex of the adaptors cIAP1, cIAP2, TRAF2 and TRAF3 and the kinase NIK. Nat Immunol.

[53] Vallabhapurapu S, Matsuzawa A, Zhang W, Tseng PH, Keats JJ, Wang H, Vignali DA, Bergsagel PL, Karin M (2008). Nonredundant and complementary functions of TRAF2 and TRAF3 in a ubiquitination cascade that activates NIK-dependent alternative NF-kappaB signaling. Nat Immunol.

[54] Du Z, Yan D, Li Z, Gu J, Tian Y, Cao J, Tang Z (2020). Genes involved in the PD-L1 pathway might associate with radiosensitivity of patients with gastric cancer. J Oncol.

[55] Ranjbar R, Hesari A, Ghasemi F, Sahebkar A (2018). Expression of microRNAs and IRAK1 pathway genes are altered in gastric cancer patients with *Helicobacter pylori* infection. J Cell Biochem.

[56] Yang M, Jin M, Li K, Liu H, Yang X, Zhang X, Zhang B, Gong A, Bie Q (2020). TRAF6 promotes gastric cancer cell self-renewal, proliferation, and migration. Stem Cells Int.

[57] Hrdinka M, Yabal M (2019). Inhibitor of apoptosis proteins in human health and disease. Genes Immun.

[58] Saleem M, Qadir MI, Perveen N, Ahmad B, Saleem U, Irshad T, Ahmad B (2013). Inhibitors of apoptotic proteins: new targets for anticancer therapy. Chem Biol Drug.

[59] Abbas R, Larisch S (2020). Targeting XIAP for promoting cancer cell death-the story of ARTS and SMAC. Cells.

[60] Kocab AJ, Duckett CS (2016). Inhibitor of apoptosis proteins as intracellular signaling intermediates. FEBS J.

[61] Peltzer N, Darding M, Walczak H (2016). Holding RIPK1 on the ubiquitin leash in TNFR1 signaling. Trends Cell Biol.

[62] Annibaldi A, Wicky John S, Vanden Berghe T, Swatek KN, Ruan J, Liccardi G, Bianchi K, Elliott PR, Choi SM, Van Coillie S, Bertin J, Wu H, Komander D, Vandenabeele P, Silke J, Meier P (2018). Ubiquitin-mediated regulation of RIPK1 kinase activity independent of IKK and MK2. Mol Cell.

[63] Moulin M, Anderton H, Voss AK, Thomas T, Wong WW, Bankovacki A, Feltham R, Chau D, Cook WD, Silke J, Vaux DL (2012). IAPs limit activation of RIP kinases by TNF receptor 1 during development. EMBO J.

[64] Sagaert X, De Wolf-Peeters C, Noels H, Baens M (2007). The pathogenesis of MALT lymphomas: where do we stand?. Leukemia.

[65] Iwamuro M, Takenaka R, Nakagawa M, Moritou Y, Saito S, Hori S, Inaba T, Kawai Y, Toyokawa T, Tanaka T, Yoshino T, Okada H (2017). Management of gastric mucosa-associated lymphoid tissue lymphoma in patients with extra copies of the MALT1 gene. World J Gastroenterol.

[66] Hu S, Du MQ, Park SM, Alcivar A, Qu L, Gupta S, Tang J, Baens M, Ye H, Lee TH, Marynen P, Riley JL, Yang X (2016). cIAP2 is a ubiquitin protein ligase for BCL10 and is dysregulated in mucosa-associated lymphoid tissue lymphomas. J Clin Invest.

[67] Maubach G, Sokolova O, Wolfien M, Rothkötter H-J, Naumann M (2013). Ca2+/calmodulin-dependent kinase II contributes to inhibitor of nuclear factor-kappa B kinase complex activation in Helicobacter pylori infection. Int J Cancer.

[68] Li Z, Chen J, Chan KW, Qiao L, Wong BC (2011). A possible role of cIAP2 in Helicobacter pylori-associated gastric cancer. Cancer Lett.

[69] Kim MA, Lee HE, Lee HS, Yang HK, Kim WH (2011). Expression of apoptosis-related proteins and its clinical implication in surgically resected gastric carcinoma. Virchows Arch.

[70] Gao X, Zhang L, Wei Y, Yang Y, Li J, Wu H, Yin Y (2019). Prognostic value of XIAP level in patients with various cancers: a systematic review and meta-analysis. J Cancer.

[71] Chang CS, Chen WN, Lin HH, Wu CC, Wang CJ (2004). Increased oxidative DNA damage, inducible nitric oxide synthase, nuclear factor kappaB expression and enhanced antiapoptosis-related proteins in Helicobacter pylori-infected non-cardiac gastric adenocarcinoma. World J Gastroenterol.

[72] Yoon H, Kim SG, Kim BK, Shin E, Kim N, Lee HJ, Kang GH, Jung HC (2017). Helicobacter pylori eradication downregulates cellular inhibitor of apoptosis protein 2 in gastric carcinogenesis. Gut Liver.

[73] Valenzuela M, Pérez-Pérez G, Corvalán AH, Carrasco G, Urra H, Bravo D, Toledo H, Quest AF (2010). Helicobacter pylori-induced loss of the inhibitor-of-apoptosis protein survivin is linked to gastritis and death of human gastric cells. J Infect Dis.

[74] Cheng XJ, Lin JC, Ding YF, Zhu L, Ye J, Tu SP (2016). Survivin inhibitor YM155 suppresses gastric cancer xenograft growth in mice without affecting normal tissues. Oncotarget.

[75] Maeda S, Yoshida H, Mitsuno Y, Hirata Y, Ogura K, Shiratori Y, Omata M (2002). Analysis of apoptotic and antiapoptotic signalling pathways induced by *Helicobacter pylori*. Gut.

[76] Yanai A, Hirata Y, Mitsuno Y, Maeda S, Shibata W, Akanuma M, Yoshida H, Kawabe T, Omata M (2003). *Helicobacter pylori* induces antiapoptosis through buclear factor-kappaB activation. J Infect Dis.

[77] Lim MCC, Maubach G, Sokolova O, Feige MH, Diezko R, Buchbinder J, Backert S, Schlüter D, Lavrik IN, Naumann M (2017). Pathogen-induced ubiquitin-editing enzyme A20 bifunctionally shuts off NF-κB and caspase-8-dependent apoptotic cell death. Cell Death Differ.

[78] Cover TL, Krishna US, Israel DA, Peek RM (2003). Induction of gastric epithelial cell apoptosis by *Helicobacter pylori* vacuolating cytotoxin. Cancer Res.

[79] Palrasu M, Zaika E, El-Rifai W, Garcia-Buitrago M, Piazuelo MB, Wilson KT, Peek RM, Zaika AI (2020). Bacterial CagA protein compromises tumor suppressor mechanisms in gastric epithelial cells. J Clin Invest.

[80] Ng VH, Hang BI, Sawyer LM, Neitzel LR, Crispi EE, Rose KL, Popay TM, Zhong A, Lee LA, Tansey WP, Huppert S, Lee E (2018). Phosphorylation of XIAP at threonine 180 controls its activity in Wnt signaling. J Cell Sci.

[81] Franco AT, Israel DA, Washington MK, Krishna U, Fox JG, Rogers AB, Neish AS, Collier-Hyams L, Perez-Perez GI, Hatakeyama M, Whitehead R, Gaus K, O'Brien DP, Romero-Gallo J, Peek RM (2005). Activation of beta-catenin by carcinogenic Helicobacter pylori. Proc Natl Acad Sci USA.

[82] Sokolova O, Bozko PM, Naumann M (2008). *Helicobacter pylori* suppresses glycogen synthase kinase 3beta to promote beta-catenin activity. J Biol Chem.

[83] Fenoglio-Preiser CM, Wang J, Stemmermann G, Noffsinger A (2003). TP53 and gastric carcinoma:a review. Hum Mutat.

[84] Joruiz SM, Beck JA, Horikawa I, Harris CC (2020). The Δ133p53 isoforms, tuners of the p53 pathway. Cancers (Basel).

[85] Mukhopadhyay D, Riezman H (2007). Proteasome-independent functions of ubiquitin in endocytosis and signaling. Science.

[86] Li W, Peng X, Lang J, Xu C (2020). Targeting mouse double minute 2: current concepts in DNA damage repair and therapeutic approaches in cancer. Front Pharmacol.

[87] Oren M, Damalas A, Gottlieb T, Michael D, Taplick J, Leal JF, Maya R, Moas M, Seger R, Taya Y, Ben-Ze'ev A (2002). Regulation of p53: intricate loops and delicate balances. Biochem Pharmacol.

[88] Shibata A, Parsonnet J, Longacre TA, Garcia MI, Puligandla B, Davis RE, Vogelman JH, Orentreich N, Habel LA (2002). CagA status of Helicobacter pylori infection and p53 gene mutations in gastric adenocarcinoma. Carcinogenesis.

[89] Gobbo César AC, de Freitas CM, Cury PM, Caetano A, Borim AA, Silva AE (2006). Genetic alterations in benign lesions: chronic gastritis and gastric ulcer. World J Gastroenterol.

[90] Kodama M, Fujioka T, Murakami K, Okimoto T, Sato R, Watanabe K, Nasu MJ (2005). Eradication of *Helicobacter pylori* reduced the immunohistochemical detection of p53 and MDM2 in gastric mucosa. Gastroenterol Hepatol.

[91] Yang Z, Shu X, Chen L, Chen J, Xie Y, Lu NH (2012). Expression of p53-MDM2 feedback loop related proteins in different gastric pathologies in relation to *Helicobacter pylori* infection: implications in gastric carcinogenesis. Clin Res Hepatol Gastroenterol.

[92] Shu X, Yang Z, Li ZH, Chen L, Zhou XD, Xie Y, Lu NH (2015). *Helicobacter pylori*infectionactivates the Akt-Mdm2-p53 signaling pathway in gastric epithelial cells. Dig Dis Sci.

[93] Wei J, Nagy TA, Vilgelm A, Zaika E, Ogden SR, Romero-Gallo J, Piazuelo MB, Correa P, Washington MK, El-Rifai W, Peek RM, Zaika A (2010). Regulation of p53 tumor suppressor by *Helicobacter pylori* in gastric epithelial cells. Gastroenterology.

[94] Bhardwaj V, Noto JM, Wei J, Andl C, El-Rifai W, Peek RM, Zaika AI (2015). Helicobacter pylori bacteria alter the p53 stress response via ERK-HDM2 pathway. Oncotarget.

[95] Wei J, Noto JM, Zaika E, Romero-Gallo J, Piazuelo MB, Schneider B, El-Rifai W, Correa P, Peek RM, Zaika AI (2015). Bacterial CagA protein induces degradation of p53 protein in a p14ARF-dependent manner. Gut.

[96] Horvat A, Noto JM, Ramatchandirin B, Zaika E, Palrasu M, Wei J, Schneider BG, El-Rifai W, Peek RM, Zaika AI (2018). *Helicobacter pylori* pathogen regulates p14ARF tumor suppressor and autophagy in gastric epithelial cells. Oncogene.

[97] Ye F, Tang C, Shi W, Qian J, Xiao S, Gu M, Dang Y, Liu J, Chen Y, Shi R, Zhang GA (2015). MDM2-dependent positive-feedback loop is involved in inhibition of miR-375 and miR-106b induced by *Helicobacter pylori* lipopolysaccharide. Int J Cancer.

[98] Wan Y, Wu W, Yin Z, Guan P, Zhou B (2011). MDM2 SNP309, gene-gene interaction, and tumor susceptibility: an updated meta-analysis. BMC Cancer.

[99] Moradi MT, Salehi Z, Asl SF, Aminian K, Hashtchin AR (2013). *Helicobacter pylori* infection and MDM2 SNP309 association with gastric cancer susceptibility. Genet Test Mol Biomarkers.

[100] Shen W, Hu P, Cao JQ, Liu XX, Shao JH (2014). MDM2 oncogene, E3 ubiquitin protein ligase T309G polymorphism and risk of oesophageal or gastric cancer: meta-analysis of 15 studies. J Int Med Res.

[101] Pan X, Li Y, Feng J, Wang X, Hao B, Shi R, Zhang G (2013). A functional polymorphism T309G in MDM2 gene promoter, intensified by Helicobacter pylori lipopolysaccharide, is associated with both an increased susceptibility and poor prognosis of gastric carcinoma in Chinese patients. BMC Cancer.

[102] Chen B, Cao L, Hu KW, Zhang JW, Meng XL, Xiong MM (2014). MDM2 SNP309 is an ethnicity-dependent risk factor for digestive tract cancers. Tumour Biol.

[103] Tongtawee T, Dechsukhum C, Leeanansaksiri W, Kaewpitoon S, Kaewpitoon N, Loyd RA, Matrakool L, Panpimanmas S (2015). Genetic polymorphism of MDM2 SNP309 in patients with helicobacter pylori-associated gastritis. Asian Pac J Cancer Prev.

[104] Shembade N, Pujari R, Harhaj NS, Abbott DW, Harhaj EW (2011). The kinase IKKalpha inhibits activation of the transcription factor NF-kappaB by phosphorylating the regulatory molecule TAX1BP1. Nat Immunol.

[105] Wertz IE, O'Rourke KM, Zhou H, Eby M, Aravind L, Seshagiri S, Wu P, Wiesmann C, Baker R, Boone DL, Ma A, Koonin EV, Dixit VM (2004). De-ubiquitination and ubiquitin ligase domains of A20 downregulate NF-kappaB signalling. Nature.

[106] Malynn BA, Ma A (2019). A20: a multifunctional tool for regulating immunity and preventing disease. Cell Immunol.

[107] Shembade N, Ma A, Harhaj EW (2010). Inhibition of NF-B signaling by A20 through disruption of ubiquitin enzyme complexes. Science.

[108] Hutti JE, Turk BE, Asara JM, Ma A, Cantley LC, Abbott DW (2007). IkappaB kinase beta phosphorylates the K63 deubiquitinase A20 to cause feedback inhibition of the NF-kappaB pathway. Mol Cell Biol.

[109] Hyeon J, Lee B, Shin SH, Yoo HY, Kim SJ, Kim WS, Park WY, Ko YH (2018). Targeted deep sequencing of gastric marginal zone lymphoma identified alterations of TRAF3 and TNFAIP3 that were mutually exclusive for MALT1 rearrangement. Mod Pathol.

[110] Maeda S, Otsuka M, Hirata Y, Mitsuno Y, Yoshida H, Shiratori Y, Masuho Y, Muramatsu M, Seki N, Omata M (2001). cDNA microarray analysis of Helicobacter pylori-mediated alteration of gene expression in gastric cancer cells. Biochem Biophys Res Commun.

[111] Sun F, Ni Y, Zhu H, Fang J, Wang H, Xia J, Ding F, Shen H, Shao S (2018). microRNA-29a-3p, up-regulated in human gastric cells and tissues with *H. Pylori* infection, promotes the migration of GES-1 Cells via A20-mediated EMT pathway. Cell Physiol Biochem.

[112] Ordureau A, Smith H, Windheim M, Peggie M, Carrick E, Morrice N, Cohen P (2008). The IRAK-catalysed activation of the E3 ligase function of Pellino isoforms induces the Lys63-linked polyubiquitination of IRAK1. Biochem J.

[113] Medvedev AE, Murphy M, Zhou H, Li X (2015). E3 ubiquitin ligases Pellinos as regulators of pattern recognition receptor signaling and immune responses. Immunol Rev.

[114] Choi SW, Park HH, Kim S, Chung JM, Noh HJ, Kim SK, Song HK, Lee CW, Morgan MJ, Kang HC, Kim YS (2018). PELI1 selectively targets kinase-active RIP3 for ubiquitylation-dependent proteasomal degradation. Mol Cell.

[115] Smith SM, Freeley M, Moynagh PN, Kelleher DP (2017). Differential modulation of *Helicobacter pylori* lipopolysaccharide-mediated TLR2 signaling by individual Pellino proteins. Helicobacter.

[116] Mao JH, Kim IJ, Wu D, Climent J, Kang HC, DelRosario R, Balmain A (2008). FBXW7 targets mTOR for degradation and cooperates with PTEN in tumor suppression. Science.

[117] Milne AN, Leguit R, Corver WE, Morsink FH, Polak M, de Leng WW, Carvalho R, Offerhaus GJ (2010). Loss of CDC4/FBXW7 in gastric carcinoma. Cell Oncol.

[118] Busino L, Millman SE, Scotto L, Kyratsous CA, Basrur V, O'Connor O, Hoffmann A, Elenitoba-Johnson KS, Pagano M (2012). Fbxw7alpha- and GSK3-mediated degradation of p100 is a pro-survival mechanism in multiple myeloma. Nat Cell Biol.

[119] Babaei-Jadidi R, Li N, Saadeddin A, Spencer-Dene B, Jandke A, Muhammad B, Ibrahim EE, Muraleedharan R, Abuzinadah M, Davis H, Lewis A, Watson S, Behrens A, Tomlinson I, Nateri AS (2011). FBXW7 influences murine intestinal homeostasis and cancer, targeting Notch, Jun, and DEK for degradation. J Exp Med.

[120] Cao J, Ge MH, Ling ZQ (2016). Fbxw7 tumor suppressor: a vital regulator contributes to human tumorigenesis. Medicine (Baltimore).

[121] Sailo BL, Banik K, Girisa S, Bordoloi D, Fan L, Halim CE, Wang H, Kumar AP, Zheng D, Mao X, Sethi G, Kunnumakkara AB (2019). FBXW7 in cancer: What has been unraveled thus far?. Cancers (Basel).

[122] Sionov RV, Netzer E, Shaulian E (2013). Differential regulation of FBXW7 isoforms by various stress stimuli. Cell Cycle.

[123] Calcagno DQ, Freitas VM, Leal MF, de Souza CR, Demachki S, Montenegro R, Assumpção PP, Khayat AS, Smith Mde A, dos Santos AK, Burbano RR (2013). MYC, FBXW7 and TP53 copy number variation and expression in gastric cancer. BMC Gastroenterol.

[124] Gong J, Cui Z, Li L, Ma Q, Wang Q, Gao Y, Sun H (2015). MicroRNA-25 promotes gastric cancer proliferation, invasion, and migration by directly targeting F-box and WD-40 Domain Protein 7, FBXW7. Tumour Biol.

[125] Zhou X, Jin W, Jia H, Yan J, Zhang G (2015). MiR-223 promotes the cisplatin resistance of human gastric cancer cells via regulating cell cycle by targeting FBXW7. J Exp Clin Cancer Res.

[126] Eto K, Iwatsuki M, Watanabe M, Ishimoto T, Ida S, Imamura Y, Iwagami S, Baba Y, Sakamoto Y, Miyamoto Y, Yoshida N, Baba H (2015). The sensitivity of gastric cancer to trastuzumab is regulated by the miR-223/FBXW7 pathway. Int J Cancer.

[127] Zebisch M, Jones EY (2015). ZNRF3/RNF43–a direct linkage of extracellular recognition and E3 ligase activity to modulate cell surface signalling. Prog Biophys Mol Biol.

[128] Hao HX, Jiang X, Cong F (2016). Control of Wnt receptor turnover by R-spondin-ZNRF3/RNF43 signaling module and its dysregulation in cancer. Cancers (Basel).

[129] Koo BK, Spit M, Jordens I, Low TY, Stange DE, van de Wetering M, van Es JH, Mohammed S, Heck AJ, Maurice MM, Clevers H (2012). Tumour suppressor RNF43 is a stem-cell E3 ligase that induces endocytosis of Wnt receptors. Nature.

[130] Park HB, Kim JW, Baek KH (2020). Regulation of Wnt signaling through ubiquitination and deubiquitination in cancers. Int J Mol Sci.

[131] Ooi CH, Ivanova T, Wu J, Lee M, Tan IB, Tao J, Ward L, Koo JH, Gopalakrishnan V, Zhu Y, Cheng LL, Lee J, Rha SY, Chung HC, Ganesan K, So J, Soo KC, Lim D, Chan WH, Wong WK, Bowtell D, Yeoh KG, Grabsch H, Boussioutas A, Tan P (2009). Oncogenic pathway combinations predict clinical prognosis in gastric cancer. PLoS Genet.

[132] Min BH, Hwang J, Kim NK, Park G, Kang SY, Ahn S, Ahn S, Ha SY, Lee YK, Kushima R, Van Vrancken M, Kim MJ, Park C, Park HY, Chae J, Jang SS, Kim SJ, Kim YH, Kim JI, Kim KM (2016). Dysregulated Wnt signalling and recurrent mutations of the tumour suppressor RNF43 in early gastric carcinogenesis. J Pathol.

[133] Cho J, Ahn S, Son DS, Kim NK, Lee KW, Kim S, Lee J, Park SH, Park JO, Kang WK, An JY, Choi MG, Lee JH, Sohn TS, Bae JM, Kim S, Kim KM (2019). Bridging genomics and phenomics of gastric carcinoma. Int J Cancer.

[134] Gao Y, Cai A, Xi H, Li J, Xu W, Zhang Y, Zhang K, Cui J, Wu X, Wei B, Chen L (2017). Ring finger protein 43 associates with gastric cancer progression and attenuates the stemness of gastric cancer stem-like cells via the Wnt-β/catenin signaling pathway. Stem Cell Res Ther.

[135] Neumeyer V, Vieth M, Gerhard M, Mejías-Luque R (2019). Mutated Rnf43 aggravates helicobacter pylori-induced gastric pathology. Cancers (Basel).

[136] Neumeyer V, Brutau-Abia A, Allgäuer M, Pfarr N, Weichert W, Falkeis-Veits C, Kremmer E, Vieth M, Gerhard M, Mejías-Luque R (2020). Loss of RNF43 function contributes to gastric carcinogenesis by impairing DNA damage response. Cell Mol Gastroenterol Hepatol.

[137] Ogawa M, Mizugishi K, Ishiguro A, Koyabu Y, Imai Y, Takahashi R, Mikoshiba K, Aruga J (2008). Rines/RNF180, a novel RING finger gene-encoded product, is a membrane-bound ubiquitin ligase. Genes Cells.

[138] Cheung KF, Lam CN, Wu K, Ng EK, Chong WW, Cheng AS, To KF, Fan D, Sung JJ, Yu J (2012). Characterization of the gene structure, functional significance, and clinical application of RNF180, a novel gene in gastric cancer. Cancer.

[139] Han F, Sun LP, Liu S, Xu Q, Liang QY, Zhang Z, Cao HC, Yu J, Fan DM, Nie YZ, Wu KC, Yuan Y (2016). Promoter methylation of RNF180 is associated with *H. pylori* infection and serves as a marker for gastric cancer and atrophic gastritis. Oncotarget.

[140] Siswanto FM, Jawi IM, Kartiko BH (2018). The role of E3 ubiquitin ligase seven in absentia homolog in the innate immune system: an overview. Vet World.

[141] Lipkowitz S, Weissman AM (2011). RINGs of good and evil: RING finger ubiquitin ligases at the crossroads of tumour suppression and oncogenesis. Nat Rev Cancer.

[142] Wong CS, Möller A (2013). Siah: a promising anticancer target. Cancer Res.

[143] Das L, Kokate SB, Rath S, Rout N, Singh SP, Crowe SE, Mukhopadhyay AK, Bhattacharyya A (2016). ETS2 and Twist1 promote invasiveness of Helicobacter pylori-infected gastric cancer cells by inducing Siah2. Biochem J.

[144] Kokate SB, Dixit P, Das L, Rath S, Roy AD, Poirah I, Chakraborty D, Rout N, Singh SP, Bhattacharyya A (2018). Acetylation-mediated Siah2 stabilization enhances PHD3 degradation in Helicobacter pylori-infected gastric epithelial cancer cells. FASEB J.

[145] Torrino S, Visvikis O, Doye A, Boyer L, Stefani C, Munro P, Bertoglio J, Gacon G, Mettouchi A, Lemichez E (2011). The E3 ubiquitin-ligase HACE1 catalyzes the ubiquitylation of active Rac1. Dev Cell.

[146] Tang D, Xiang Y, De Renzis S, Rink J, Zheng G, Zerial M, Wang Y (2011). The ubiquitin ligase HACE1 regulates Golgi membrane dynamics during the cell cycle. Nat Commun.

[147] Ugarteburu O, Sánchez-Vilés M, Ramos J, Barcos-Rodríguez T, Garrabou G, García-Villoria J, Ribes A, Tort F (2020). Physiopathological bases of the disease caused by HACE1 mutations: alterations in autophagy, mitophagy and oxidative stress response. J Clin Med.

[148] Li JC, Chang X, Chen Y, Li XZ, Zhang XL, Yang SM, Hu CJ, Zhang H (2019). Loss of the tumor suppressor HACE1 contributes to cancer progression. Curr Drug Targets.

[149] Sakata M, Kitamura YH, Sakuraba K, Goto T, Mizukami H, Saito M, Ishibashi K, Kigawa G, Nemoto H, Sanada Y, Hibi K (2009). Methylation of HACE1 in gastric carcinoma. Anticancer Res.

[150] Yoon JH, Kim O, Nam SW, Lee JY, Park WS (2017). NKX6.3 regulates reactive oxygen species production by suppressing NF-kB and DNMT1 activities in gastric epithelial cells. Sci Rep.

[151] Chen YL, Li DP, Jiang HY, Yang Y, Xu LL, Zhang SC, Gao H (2018). Overexpression of HACE1 in gastric cancer inhibits tumor aggressiveness by impeding cell proliferation and migration. Cancer Med.

[152] Wang M, Dai W, Ke Z, Li Y (2020). Functional roles of E3 ubiquitin ligases in gastric cancer. Oncol Lett.

[153] Endo S, Yamato K, Hirai S, Moriwaki T, Fukuda K, Suzuki H, Abei M, Nakagawa I, Hyodo I (2011). Potent in vitro and in vivo antitumor effects of MDM2 inhibitor nutlin-3 in gastric cancer cells. Cancer Sci.

[154] Lan H, Tang Z, Jin H, Sun Y (2016). Neddylation inhibitor MLN4924 suppresses growth and migration of human gastric cancer cells. Sci Rep.

[155] Yi H, Yan X, Luo Q, Yuan L, Li B, Pan W, Zhang L, Chen H, Wang J, Zhang Y, Zhai Y, Qiu MZ, Yang DJ (2018). A novel small molecule inhibitor of MDM2-p53 (APG-115) enhances radiosensitivity of gastric adenocarcinoma. J Exp Clin Cancer Res.

[156] Park J, Cho J, Song EJ (2020). Ubiquitin-proteasome system (UPS) as a target for anticancer treatment. Arch Pharm Res.

[157] Zhao XY, Wang XY, Wei QY, Xu YM, Lau ATY (2020). Potency and selectivity of SMAC/DIABLO mimetics in solid tumor therapy. Cells.

[158] Morrish E, Brumatti G, Silke J (2020). Future therapeutic directions for smac-mimetics. Cells.

[159] Ma J, Cheng L, Liu H, Zhang J, Shi Y, Zeng F, Miele L, Sarkar FH, Xia J, Wang Z (2013). Genistein down-regulates miR-223 expression in pancreatic cancer cells. Curr Drug Targets.

[160] Zan L, Chen Q, Zhang L, Li X (2019). Epigallocatechin gallate (EGCG) suppresses growth and tumorigenicity in breast cancer cells by downregulation of miR-25. Bioengineered.

[161] Sárközy M, Kahán Z, Csont T (2018). A myriad of roles of miR-25 in health and disease. Oncotarget.

[162] Yang K, Wu H, Zhang Z, Leisten ED, Nie X, Liu B, Wen Z, Zhang J, Cunningham MD, Tang W (2020). Development of selective histone deacetylase 6 (HDAC6) degraders recruiting Von Hippel-Lindau (VHL) E3 ubiquitin ligase. ACS Med Chem Lett.

[163] Zheng S, Tao W (2020). Targeting Cullin-RING E3 ligases for radiosensitization: from NEDDylation inhibition to PROTACs. Front Oncol.

[164] Wang B, Wu S, Liu J, Yang K, Xie H, Tang W (2019). Development of selective small molecule MDM2 degraders based on nutlin. Eur J Med Chem.

[165] Fang Y, Liao G, Yu B (2020). Small-molecule MDM2/X inhibitors and PROTAC degraders for cancer therapy: advances and perspectives. Acta Pharm Sin B.

